# Design of metacontinua in the aeroacoustic spacetime

**DOI:** 10.1038/s41598-020-74304-5

**Published:** 2020-10-23

**Authors:** Umberto Iemma, Giorgio Palma

**Affiliations:** Department of Engineering, Università degli Studi Roma Tre, via Vito Volterra 62, 00146 Rome, Italy

**Keywords:** Acoustics, Aerospace engineering, Mechanical engineering

## Abstract

The effect of background flows on the response of acoustic metamaterials is a key aspect that prevented the full disclosure of their potential in those applications where an aerodynamic velocity field strongly influences the propagation of acoustic disturbances. Indeed, the classic approaches for metamaterial design do not consider the aeroacoustic interaction, and the resulting metamaterials cannot preserve their response when operating in flows. So far, only few authors have addressed the problem, mostly focusing on understanding the phenomenon or identifying corrective techniques with limited usability in practical applications. The present study proposes a general method for the modification of the mechanical properties of acoustic metacontinua to preserve their response in presence of a background flow. The method is based on the application of spacetime coordinate transformations exploiting the spacetime formal invariance of the generalised d’Alembertian. This methodology applies to the equation governing the propagation of acoustic disturbances in a metamaterial having arbitrary constitutive equations independently on the method used for its original design. The approach is validated through numerical simulations, using as a benchmark the problem of the acoustic cloaking of a cylinder impinged by a perturbation generated by an isotropic point source within a flowing medium. Numerical results are obtained for an asymptotic Mach number $M_\infty \le 0.35$.

## Introduction

**Figure 1:**
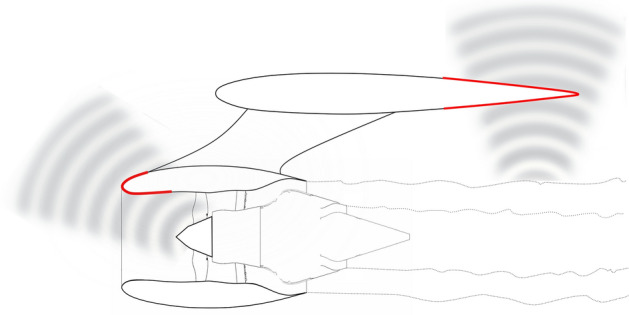
Qualitative illustration of acoustic cloaking applications for the mitigation of aircraft community noise. Acoustic invisibility of specific components may reduce the noise reflected towards the ground.

After their groundbreaking advent in the first decade of the century (see Cummer and Schurig^1^), acoustic metamaterials have become one of the most investigated topics in applied physics and engineering. The application potential of acoustic metamaterials has triggered the interest of the research community, producing a large number of concepts of remarkable interest in many fields of engineering. Although the readiness level of the associated technologies is still far to be of some relevance in end user applications, acoustic metamaterials have captured the attention also of aeronautical industries as one of the breakthrough technologies potentially capable to cope with the noise mitigation challenge foreseen in the next three decades. A technological quantum leap is nowadays recognized to be indispensable because of the substantial development saturation of the existing technologies in a scenario of constantly growing market. The difficulties in porting the existing methods for metamaterial design to aeronautical applications are primarily related to the strong interaction of the aerodynamic field with the acoustic disturbance. From the physical point of view, the main effect of aerodynamic convection is the distortion of the wave propagation pattern according to aerodynamic transport, that makes a statically designed metamaterial fail to achieve its target response. This deterioration of the efficiency does not depend on the specific method used for the static design. On the other hand, from the mathematical viewpoint, the presence of a background flow changes the structure of the governing equations introducing mixed spacetime derivatives of the unknown function. The different form of the equations makes the approaches based on formal invariance under conformal mappings fail, at least remaining within the framework of the classic approach to the mechanics of fluids. In 1981, Unruh^2^ developed the concept of acoustic analogue spacetime, having noticed that the equations governing the propagation of an acoustic perturbation in a moving medium exhibit a relativistic structure. In the following years, the concept has been further developed by Visser^3,4^ and Unruh^5^ and exhaustively formalised, for a barotropic fluid, in Visser and Molina–Paris^6^ making use of the analytical tools of the Lorentzian differential geometry. This relativistic reinterpretation of the aeroacoustic equations has represented the foundation of the innovative and prolific spacetime reformulation of the acoustic metamaterials models, introduced to circumvent the limitations of the existing approaches. Garcia–Meca et al.^7–9^ applied the concept of analogue spacetimes to develop the Analogue Transformation Approach (ATA) as an evolution of the Standard Transformation Approach (STA), capable to deal with a background aerodynamic flow. The approach extends to generalized acoustics the design methodology based on conformal mappings introduced by Pendry, Schurig, and Smith^10^ and Leonhardt^11^ in the electromagnetism domain. Although the method is of general applicability (the only assumptions is that the relevant wave equation can be rewritten in the form of massless Klein–Gordon equation), its actual use is subordinate to the existence of a suitable mapping, which is typically available only for very simple geometries of the domain, and is of limited usability in realistic aeronautical applications. Indeed, the geometrical constraints imposed to most of the constructive elements of an aircraft by the aerodynamic functionality make impossible the use of fairings or coverings of simple shape just to modify the acoustics signature. Figure 1 depicts how the acoustic cloaking concept can be applied to engines nacelles and wings to reduce the noise reflected towards the ground. It appears clearly how the peculiar geometry of the treated components is dictated by their aerodynamic function. In principle, quasi-conformal transformations could be obtained numerically for arbitrary geometries at the cost of a significant computational burden. Nevertheless, it has been demonstrated in Iemma and Palma^12^ that the effectiveness of numerically-generated transformations strongly depends on the size of the metamaterial device, thus reducing significantly the applicability of the method. Approaches based on the Doppler correction of the static metamaterial design has been presented in Huang, Zhong and Stalnov^13^ and Iemma^14^, whereas detailed analyses of the effects of the properties of the background aerodynamic field have been published by Ryoo and Jeon^15^. More recently, He, Zhong and Huang^16^ presented a method for the design of arbitrarily-shaped cloaks operating in a flow, based on the high-order Born’s approximation of the governing equation and numerical optimization. To the authors’ knowledge, this paper represents the most advanced contribution to the problem of scattering cancellation in realistic applications of aeronautical or maritime engineering.

The method presented here is also based on the spacetime reformulation of the problem and uses the same analytical tools of the ATA approach, but in a different fashion. First, the equation governing the propagation of an acoustic perturbation in a generic metafluid is rewritten in the acoustic spacetime. Then, its spacetime metric, which depends on the mechanical properties of the material, is corrected by means of spacetime transformations capable to recast the convective wave equation in the form of the standard wave equation. Finally, the components of the corrected metric are reinterpreted as the mechanical properties of the metacontinuum required to operate efficiently in a flow. The transformations used are the *Prandtl–Glauert* and the *Taylor* transformations, which include the effects of a uniform and non-uniform background flows, respectively. These transformations are widely used in the aerodynamic and aeroacoustic communities to take into account the effects of compressibility and convection under certain specific assumptions (see, e.g. Mancini et al.^17^ or Gennaretti et al.^18^). It is worth noting that, although conceptually related to Lorentz boosts, these transformations are not *Lorentzian*, *i.e.*, they have lost their capacity to preserve the length of a spacetime element of arc, due to their peculiar structure. A detailed discussion on this point is presented in Gregory et al.^19^.

## Results

### The acoustic spacetime for metafluids

The propagation of an acoustic perturbation in a standard moving medium is governed by the generalised spacetime d’Alembertian for the acoustic potential $\varphi$. Indicating with $\mathbf{v}=\nabla \Phi$ the potential aerodynamic velocity, the equation governing the propagation of an acoustic disturbance in the moving medium is

1 $$\begin{aligned} -{\partial _{t} {} }\left[ \frac{\varrho _{{0}}}{c_{{0}}^2}\left( {\partial _{t} {\varphi } }+\mathbf{v}\cdot \nabla \varphi \right) \right] +\nabla \cdot \left[ \varrho _{{0}}\,\nabla \varphi - \frac{\varrho _{{0}}}{c_{{0}}^2}\mathbf{v}\left( {\partial _{t} {\varphi } }+\mathbf{v}\cdot \nabla \varphi \right) \right] =S \end{aligned}$$

where *S* represents all the noise sources, and $\varrho _{{0}}(t,\varvec{x})$ and $c_{{0}}(t,\varvec{x})$ are the local values of density and speed of sound, related to the aerodynamic state of the fluid through the non-linear Bernoulli’s theorem (see Supplementary Information). Equation (1) fully describes the propagation of an acoustic disturbance in presence of flow, provided that no sources of sound induced by vorticity or entropy fluctuations are present. The correction method proposed here starts from the reformulation of the problem in the four-dimensional spacetime where, as first noticed by Unruh^5^ and Visser^4^, Eq. (1) exhibits a Lorentzian structure. The coordinates of a spacetime *event* are collected in the four-vector ${\varvec{\xi }}\equiv \left( \xi _0,\xi _1,\xi _2,\xi _3\right) \equiv \left( c_{\mathsf{ref}}\,t,x_1,x_2,x_3\right)$. In the following, we will choose as reference speed of sound the value $c_\infty$ at infinite distance from the obstacle, adopting units such that $c_\infty =1$. Equation (1) can be recast in the form of generalised, spacetime d’Alembertian

2 $$\begin{aligned} \frac{1}{\sqrt{-g}}\varvec{\partial }\cdot \left( {\sqrt{-g}}\,\varvec{g}^{-1}\varvec{\partial }\varphi \right) =S \end{aligned}$$

where $\varvec{\partial }=(\partial _{\mathrm{0}},\partial _{\mathrm{1}},\partial _{\mathrm{2}},\partial _{\mathrm{3}})$. The metric depends on the background flow as

3 $$\begin{aligned} \varvec{g}=\frac{\varrho _{{0}}}{c_{{0}}}\left( \begin{array}{ccc} -(c_{{0}}^{{2}}- \text {v}^2) &{} \vdots &{}-\mathbf{v}^T \\ \cdots &{} \cdot &{} \cdots \\ -\mathbf{v}&{} \vdots &{} \mathbf{I}\end{array}\right) , \;\; \varvec{g}^{-1}=\frac{1}{\varrho _{{0}}c_{{0}}}\left( \begin{array}{ccc} -1 &{} \vdots &{}-\mathbf{v}^T \\ \cdots &{} \cdot &{} \cdots \\ -\mathbf{v}&{} \vdots &{} c_{{0}}^{{2}}\mathbf{I}-\mathbf{v}\otimes \mathbf{v}\end{array}\right) \end{aligned}$$

**Figure 2:**
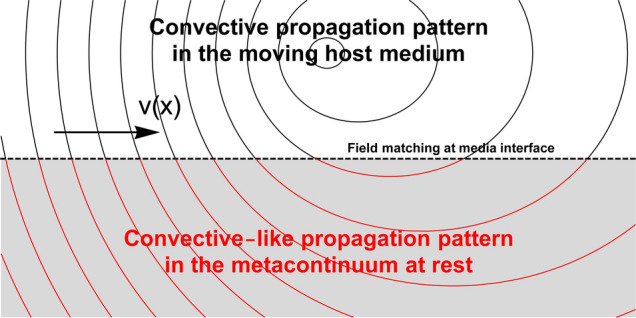
Pictorial representation of the matching of the propagation pattern at the interface between the moving hosting medium and the metafluid at rest.

The corresponding governing equation for a generic metacontinuum can be derived by recasting in spacetime form the general formulation introduced by Norris^20,21^. The most general form of equation governing the propagation of an acoustic disturbance within a metafluid can be written as (see Supplementary Information)

4 $$\begin{aligned} - {\partial _{tt} {p} } + {c_{\mathsf{ref}}^2}\;\hat{\mathcal {K}}\; \nabla \cdot \left( \mathbf{Q}\,\hat{\varvec{\varrho }}^{-1}\,\mathbf{Q}\;\nabla p \right) = 0 \end{aligned}$$

where $\varrho _{\mathsf{ref}}$, $\mathcal {K}_{\mathsf{ref}}$, and $c_{\mathsf{ref}}={\sqrt{\mathcal {K}_{\mathsf{ref}}/\varrho _{\mathsf{ref}}}}$ are the reference density, bulk modulus, and speed of sound, respectively. $\mathbf{Q}$ can be any symmetric tensor such that $\nabla \cdot \mathbf{Q}=0$, $\varvec{\varrho }=\hat{\varvec{\varrho }}\varrho _{\mathsf{ref}}$ represents the anysotropic inertia of the material, and the Cauchy stress tensor for such a material is given by $\varvec{\sigma }=-p\,\mathbf{Q}$. Also Eq. (4) can be rewritten in form of generalised d’Alembertian with metric

5 $$\begin{aligned} \hat{\varvec{g}}={\sqrt{\frac{\hat{\mathcal {K}}Q^2}{\hat{\varrho }}}}\left( \begin{array}{ccc} -1 &{} \vdots &{} 0 \\ \cdots &{} \cdot &{} \cdots \\ 0 &{} \vdots &{} \mathbf{Q}^{-1}\,\hat{\varvec{\varrho }}\,\mathbf{Q}^{-1} \end{array}\right) , \;\; {\hat{\varvec{g}}^{-1}}={\sqrt{\frac{\hat{\varrho }}{\hat{\mathcal {K}}Q^2}}}\left( \begin{array}{ccc} -1 &{} \vdots &{} 0 \\ \cdots &{} \cdot &{} \cdots \\ 0 &{} \vdots &{} \mathbf{Q}\,\hat{\varvec{\varrho }}^{-1}\,\mathbf{Q}\end{array}\right) \end{aligned}$$

where $Q=\det \mathbf{Q}$, $\hat{\varrho }=\det \hat{\varvec{\varrho }}$. Metrics (3) and (5) are both Lorentzian, with signatures (3, 1). The local differential properties of the spacetime manifolds associated with the two equations deserve some additional remark. As already observed, the spacetime reformulation of Eq. (1) reveals how sounds propagates onto a curved manifold, whose curvature depends on the non-uniformity of the background aerodynamics (see Visser^4^). It is interesting to notice that also Eq. (4) relies on an event-dependent metric through the mechanical properties of the metacontinuum. More specifically, $\hat{\varvec{g}}$ is a function of ${\varvec{\xi }}$ independently on the kinematic status of the metafluid particles. Starting from this observation, the objective of the present work can be interpreted as a method to identify the mechanical properties of a metacontinuum capable to match the local differential geometry of the aeroacoustic spacetime induced in the hosting medium by its own motion. In a few words, the method corrects the mechanical properties of the metacontinuum to match convective propagation patterns in the quiescent medium. The concept is depicted in Fig. 2, where the media interface is assumed to be aerodynamically (but not acoustically) impermeable. Using this approach, any device based on a metacontinuum conceived and designed to operate in static condition can be corrected to preserve its response in presence of a background flow.

### Metric correction through spacetime transformations

**Figure 3:**
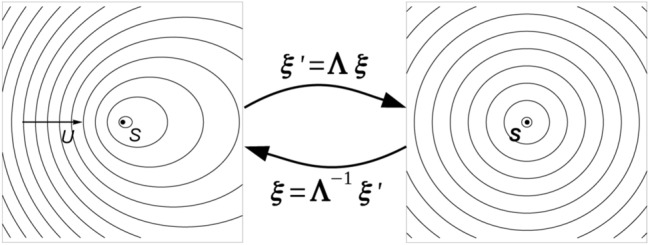
Spacetime coordinate transformation that maps a convective propagation pattern in the physical space into an isotropic one in the transformed space.

Let’s consider the transformation ${\varvec{\xi }}^\prime =\varvec{\Lambda }\,{\varvec{\xi }}$ that stretches the spacetime so as to make a convected wave recover an isotropic propagation pattern (see Fig. 3). Indicating with $\varvec{g}$ the metric of the convective wave equation, this requirement implies that $\varvec{\Lambda }$ converts $\varvec{g}$ into the Minkowski metric tensor $\varvec{\eta }$, *i.e.*,

6 $$\begin{aligned} \varvec{g}^\prime =\varvec{\Lambda }\,\varvec{g}\,\varvec{\Lambda }^T=\left( \begin{array}{ccc} -1 &{} \vdots &{} 0 \\ \cdots &{} \cdot &{} \cdots \\ 0 &{} \vdots &{} \mathbf{I}\end{array}\right) = \varvec{\eta } \end{aligned}$$

The method proposed follows three steps

1. Interpret the spacetime metric of Eq. (4), $\hat{\varvec{g}}$ as the image in the transformed space of the unknown metric $\check{\varvec{g}}$ associated to the equation governing the wave propagation inside the corrected metacontinuum *i.e.*, $\hat{\varvec{g}}=\check{\varvec{g}}^\prime =\varvec{\Lambda }\,\check{\varvec{g}}\varvec{\Lambda }^T$.
2. Obtain the unknown metric tensor $\check{\varvec{g}}$ by applying the inverse transformation, $\varvec{\Lambda }^{-1}$, to $\hat{\varvec{g}}$.
3. Write the generalised D’Alembertian for the corrected metacontinuum
  7 $$\begin{aligned} \frac{1}{\sqrt{-\check{g}}}\varvec{\partial }\cdot \left( {\sqrt{- \check{g}}}\,\check{\varvec{g}}^{-1}\,\varvec{\partial }p \right) =0 \end{aligned}$$
  and re-interpret the equation coefficients as the mechanical properties of the new material.

Unfortunately, a transformation capable to convert the metric tensor (3) is not available, and analytical forms for $\varvec{\Lambda }$ are available only for a uniform stream and for an incompressible, non-uniform potential flow. Although these assumptions for the background aerodynamics may appear to be too limiting, it must be observed that they are acceptable in many aeronautical applications, where the effects of viscosity and compressibility are small in many operating conditions. In addition, it is worth noting that for a more complex aerodynamics the approach remains valid in principle, and a suitable $\varvec{\Lambda }$ could be determined through numerical optimization, even if at the cost of a substantial computing effort. In the following, we consider the aerodynamic velocity field as a superposition of a uniform stream aligned with the $\xi _{1}$ direction and a perturbation induced by an impermeable obstacle, $\mathbf{v}(\varvec{x})=U_\infty \,\varvec{e}_{1}+\mathbf{u}(\varvec{x})$.

#### The Prandtl–Glauert transformation

The spacetime form of the *Prandtl–Glauert* transformation reads

8 $$\begin{aligned} \xi '_0 = \beta \xi _0 + M_\infty \frac{\xi _1}{\beta }, \quad \xi '_1&= \frac{\xi _1}{\beta }, \quad \xi '_2 = \xi _2, \quad \xi '_3 = \xi _3 \end{aligned}$$

or, in matrix form,

9 $$\begin{aligned} \varvec{\Lambda }_{\mathrm{PG}}=\left( \begin{array}{cccc} \beta &{}\quad M_\infty /\beta &{}\quad 0 &{}\quad 0\\ 0 &{}\quad 1/\beta &{}\quad 0 &{}\quad 0\\ 0 &{}\quad 0 &{}\quad 1 &{}\quad 0\\ 0 &{}\quad 0 &{}\quad 0 &{}\quad 1\\ \end{array}\right) \end{aligned}$$

where $M_\infty =U_\infty /c_\infty$ is the Mach number of the aerodynamic velocity $\mathbf{v}=U_\infty \,\varvec{e}_{1}$, and $\beta ={\sqrt{1-M_{\infty }^{2}}}$. The Prandtl-Glauert transformation $\varvec{\Lambda }_{\mathrm{PG}}$ can recover the metric of the static wave equation when applied to the convective wave equation for a uniform and stationary background flow (see Supplementary Information for details).The total derivative in the physical space, $D_{\mathrm{t}}^\infty = \partial _{\mathrm{t}}+\mathbf{v}\cdot \nabla =\partial _{\mathrm{t}}+U_\infty \partial _{\mathrm{1}}$, is transformed by $\varvec{\Lambda }_{\mathrm{PG}}$ into the standard time-derivative in the transformed spacetime $\partial _{\mathrm{t'}}$. Hence, the metric tensor $\varvec{g}_{\mathrm{PG}}$ such that $\varvec{\eta }=\varvec{\Lambda }_{\mathrm{PG}}\,\varvec{g}_{\mathrm{PG}}\,\varvec{\Lambda }_{\mathrm{PG}}^T$ has the form

10 $$\begin{aligned} \varvec{g}_{\mathrm{PG}}=\frac{\rho _\infty }{c_\infty }\left( \begin{array}{cccc} -(c_\infty ^2 - \text {U}^2_\infty ) &{}\quad -U_\infty &{}\quad 0 &{}\quad 0 \\ -U_\infty &{}\quad 1 &{}\quad 0 &{}\quad 0 \\ 0 &{}\quad 0 &{}\quad 1 &{}\quad 0 \\ 0 &{}\quad 0 &{}\quad 0 &{}\quad 1 \end{array}\right) , \;\; \varvec{g}_{\mathrm{PG}}^{-1}=\frac{1}{\rho _\infty c_\infty }\left( \begin{array}{cccc} -1 &{}\quad -U_\infty &{}\quad 0 &{}\quad 0 \\ -U_\infty &{}\quad c_\infty ^2 - U^2_\infty &{}\quad 0 &{}\quad 0 \\ 0 &{}\quad 0 &{}\quad c_\infty ^2 &{}\quad 0 \\ 0 &{}\quad 0 &{}\quad 0 &{}\quad c_\infty ^2 \end{array}\right) \end{aligned}$$

Considering that in Eq. (1) the velocity field can be arbitrarily complex, the application of $\varvec{\Lambda }_{\mathrm{PG}}^{{-1}}$ to the metric tensor (3) introduces only a uniform convective correction that does not take into account the contribution of the aerodynamic perturbation velocity $\mathbf{u}(\varvec{x})$. The Supplementary Information include the estimate of this effect by recasting Eq. (1) in the form of uniformly convected wave equation and isolating to the RHS all the terms depending on non-uniform flow component, $-D_{\mathrm{tt}}^\infty \varphi +\nabla ^2\varphi =\sigma _{PG}$. Detailed analyses of the order of magnitude and acoustic properties of the equivalent sources induced by non-uniformity and compressibility of the background flow are given in He, Zhong and Huang^16^ and Ryoo and Jeon^15^.

#### The Taylor transformation

The Taylor transformation, $\varvec{\Lambda }_{\mathrm{T}}$, was introduced^22^ to determine a correction of aeroacoustic measurements to take into account the effect of a low-speed, non-uniform flow on the propagation of an acoustic perturbation. The transformation assumes that the background flow is steady, incompressible, and potential, $\mathbf{v}(\varvec{x})=\nabla \Phi (\varvec{x})$ with $\nabla ^2\Phi =0$. In terms of superposition of an aerodynamic perturbation onto an asymptotic, uniform free stream, the aerodynamic velocity potential is $\Phi (\varvec{x})=U_\infty \, x_1 + \phi (\varvec{x})=U_\infty \, \xi _1 + \phi ({\varvec{\xi }})$ with $\mathbf{u}=\nabla \phi$. In addition, $M_\infty ^2<<1$. The form of the transformation is

11 $$\begin{aligned} \xi '_0&= \xi _0 + M_\infty \hat{\Phi }(\varvec{x}) \nonumber \\ \xi '_i&= \xi _i \end{aligned}$$

with $\hat{\Phi }$ = $\Phi /\Vert U_\infty \Vert$ or in matrix form

12 $$\begin{aligned} \varvec{\Lambda }_{\mathrm{T}}=\left( \begin{array}{ccc} 1 &{} \vdots &{} M_\infty \nabla \hat{\Phi }(\varvec{x}) \\ \cdots &{} \cdot &{} \cdots \\ 0 &{} \vdots &{} \mathbf{I}\end{array}\right) \end{aligned}$$

The transformation maps the $\mathcal {O}(M_\infty )$ convective wave equation in the physical space into the static d’Alembertian in the ${\varvec{\xi }}'$ spacetime. The converted metric is $\varvec{g}_{\mathrm{T}}$

13 $$\begin{aligned} \varvec{g}_{\mathrm{T}}=\frac{\rho _\infty }{c_\infty }\left( \begin{array}{ccc} -c_\infty ^2 &{} \vdots &{}-\mathbf{v}^T \\ \cdots &{} \cdot &{} \cdots \\ -\mathbf{v}&{} \vdots &{} \mathbf{I}\end{array}\right) , \;\; \varvec{g}_{\mathrm{T}}^{-1}=\frac{1}{\rho _\infty c_\infty }\left( \begin{array}{ccc} -1 &{} \vdots &{}-\mathbf{v}^T \\ \cdots &{} \cdot &{} \cdots \\ -\mathbf{v}&{} \vdots &{} c_\infty ^2\mathbf{I}\end{array}\right) + \mathcal {O}(M_\infty ^2) \end{aligned}$$

Also in this case, Eq. (1) can be rearranged to isolate in $\sigma _T$ the residual terms that under the action of $\varvec{\Lambda }_{\mathrm{T}}$ do not contribute to the wave operator in the transformed space

14 $$\begin{aligned} -\frac{1}{c_\infty ^2}\left( {\partial _{tt} {\varphi } } + 2 \mathbf{v}\cdot {\partial _{t} {\varphi } }\,\nabla \varphi \right) + \nabla ^2 \varphi = \sigma _{T} \end{aligned}$$

It should be noted that assuming a uniform background flow $\mathbf{v}=U_\infty \varvec{e}_1$, Eq. (12) becomes

15 $$\begin{aligned} \varvec{\Lambda }_{\mathrm{TUS}}=\left( \begin{array}{cccc} 1 &{}\quad M_\infty &{}\quad 0 &{}\quad 0\\ 0 &{}\quad 1 &{}\quad 0 &{}\quad 0\\ 0 &{}\quad 0 &{}\quad 1 &{}\quad 0\\ 0 &{}\quad 0 &{}\quad 0 &{}\quad 1\\ \end{array}\right) \end{aligned}$$

### Numerical assessment

The method is applied to the problem of the acoustic cloaking of a circular cylinder of radius $r_1$, impinged by the acoustic perturbation generated by an isotropic point source. The external radius of the cloaking mantle is $r_2$ and the source is located at a distance of $7.5\,r_2$ in the direction orthogonal to the incoming free stream. For each spacetime transformation, the response is assessed by evaluating the insertion loss,

16 $$\begin{aligned} IL = 10 \log _{10} \left( {p_{i}^2}/{p_{t}^2} \right) _{r_{mics}} \end{aligned}$$

along two arcs of monitoring points (*virtual microphones*) located in the near and far filed, at $r^{N}_{mics}=1.5 r_2$ and $r^{F}_{mics}=20 r_2$, respectively. Figure 5 shows the results obtained for $M_\infty =0.3$ and $k r_2=\omega \,r_2/c_\infty =4$ (dashed red line for the bare obstacle, dash-dotted blue line for the static cloak and black continuous line for the corrected cloaking). The convective designs obtained with the three spacetime transformations show a remarkable, though not perfect, recovery of the cloaking performance. The lack of a perfect correction is not surprising, and it’s essentially due to the residual terms $\sigma _{PG}$ and $\sigma _{T}$, whose exact form is derived and commented in the Supplementary Information. The overall effect of the Taylor transformation appears to be closer to a perfect correction, confirming the impact of the non-uniformity of the flow on the acoustic propagation. Indeed, $\varvec{\Lambda }_{\mathrm{PG}}$ and $\varvec{\Lambda }_{\mathrm{TUS}}$ generate near field polar pattern (Fig. 5c,e) departing from the ideal isotropic one more than what is produced by $\varvec{\Lambda }_{\mathrm{T}}$ (Fig. 5a). This effect reflects in the far field in form of ripples that are evident for $\varvec{\Lambda }_{\mathrm{PG}}$ and $\varvec{\Lambda }_{\mathrm{TUS}}$ but almost completely absent for $\varvec{\Lambda }_{\mathrm{T}}$. These considerations are confirmed in Fig. 6, which depicts the instantaneous pressure field in the region of the domain surrounding the object and the source. The effectiveness of the spacetime correction is evident for all the transformations used, with an almost perfect recovery obtained by $\varvec{\Lambda }_{\mathrm{T}}$. Some additional information is provided by the estimate of the normalised $L^2$-norm of the scattering field $p_{s}$, calculated as

17 $$\begin{aligned} L^{2}_{N} = \left. {\sqrt{\int _{\Omega _h} \left| p_{s}\right| ^2}} \bigg /{\sqrt{\int _{\Omega _h} \left| p_{s}^{B0}\right| ^2}}\right. \end{aligned}$$

being $p_{s}^{B0}$ the scattering field around the bare obstacle in quiescent medium. The value of the norm is depicted Fig. 7 as a function of the Mach number for $k\,r_2=2$ (panel (a)) and $k\,r_2=4$ (panel (b)). A first remark that can be done concerns the entity of performance degradation of the static cloak. Its increment with Mach and frequency is evident, yielding a scattering level even higher than the untreated obstacle in the most severe case $M_\infty =0.35$, $k\,r_2=4$. Beyond this initial consideration, the plots confirm that $\varvec{\Lambda }_{\mathrm{T}}$ always outperform $\varvec{\Lambda }_{\mathrm{TUS}}$ and reveal that the $\varvec{\Lambda }_{\mathrm{PG}}$ correction is better at higher Mach, when measured over the entire domain $\Omega _h$. This effect is due to the $\mathcal {O}(M_\infty )$ approximation of the Taylor transformation. A detailed analysis of the order of magnitude and the terms neglected in Taylor transformation is given in Iemma and Palma^23^ The Supplementary Information available online report the complete set of simulations for $0\le M_\infty \le 0.35$ and $1\le k r_2 \le 4$.

## Discussion

In the present paper, the method has been applied only to the correction of a cloaking mantle, although, in principle, it’s applicable to any metamaterial designed in static conditions, regardless of the specific approach used in its derivation. The choice of the cloaking of a cylinder as reference test case derives not only from its relevance as the most widely used benchmark in the field of metamaterial science, but also from the fact that scattering cancellation represents a target response of high interest in aeronautical engineering applications, where the interaction of the acoustic disturbances with the airframe components strongly influences the perturbation that reaches the ground. The numerical simulations performed show that the proposed convective correction can recover most of the effectiveness of a metacontinuum designed to operate in static conditions. As already pointed out, the not perfect recovery of the cloaking efficiency is due to the fact that, for both transformations, the metric which is mapped onto $\varvec{\eta }$ is not equal to $\varvec{g}$. In the Supplementary Information, the residual terms derived from this difference are collected into the equivalent source terms $\sigma _{PG}$ and $\sigma _{T}$. Here, the same operation is performed directly on the spacetime d’Alembertian and expressed in terms of metrics’ difference. Noting that $g_{{\varvec{\Lambda }}}$ is event-independent for both the transformations analyzed here, Eq. 2 can be easily recast in the form

18 $$\begin{aligned} \varvec{\partial }\cdot \varvec{g}^{-1}_{{\varvec{\Lambda }}}\varvec{\partial }\varphi =S -\varvec{\partial }\cdot \left( \varvec{g}^{-1}-\varvec{g}^{-1}_{{\varvec{\Lambda }}}\right) \varvec{\partial }\varphi - \frac{1}{\sqrt{-g}}\varvec{\partial }{\sqrt{-g}}\cdot \varvec{g}^{-1}\varvec{\partial }\varphi \end{aligned}$$

where $\varvec{g}_{{\varvec{\Lambda }}}=\varvec{g}_{\mathrm{PG}}$ for the Prandtl–Glauert transformation, and $\varvec{g}_{{\varvec{\Lambda }}}=\varvec{g}_{\mathrm{T}}$ for the Taylor transformation. When one of the spacetime transformations is applied to Eq. (18), the static wave equation resulting in the mapped space is forced by additional sources generated by the effect of the transformation on the residual terms induced by the difference between the metrics $\varvec{g}_{{\varvec{\Lambda }}}$ and $\varvec{g}$. It is worth emphasizing that the method presented here is based on the correction of the metacontinuum metric tensor $\hat{\varvec{g}}$ to make its acoustic response compatible with that of a moving compressible medium where Eq. (1) holds. From this point of view, the residual terms on the RHS of Eq. (18) can be considered as the *residual wave operator* that, in the transformed spacetime, governs the acoustic field given by the difference between the one obtained with Eq. (7) and the ideal perfectly corrected field. The comparison of Eqs. (3), (10), and (13) shows how these residual terms include the effects of aerodynamic compressibility and non uniformity of the background flow for both the transformations used. The importance of these effects on the acoustic propagation in the near and far field strongly depends on the specific problem under investigation. He, Zhong and Huang^16^ and Ryoo and Jeon^15^ published two extensive analyses on how these phenomena influence the efficiency of an acoustic cloak impinged by a planar wave front. The results of the numerical simulations presented here demonstrate that the convective correction actually achieved represents, in any case, a substantial improvement of the performance of the metamaterial-based device. The performance recovery attained at $M_\infty =0.3$ is definitely satisfactory from the engineering point of view. This condition is compatible with the operations of commercial aircraft at take off and landing and discloses a virtually uncountable number of unconventional concepts for the design of innovative noise-abatement devices. Indeed, one advantage of the method is that can be applied to any existing static design, making the correction of existing concepts particularly easy. Currently, the method is being applied to the correction of the elasticity tensors obtained with the computational homogeneisation approach presented by Mao, Rumpler, and Göransson^24^ within the context of the European project AERIALIST.

## Methods

**Figure 4:**
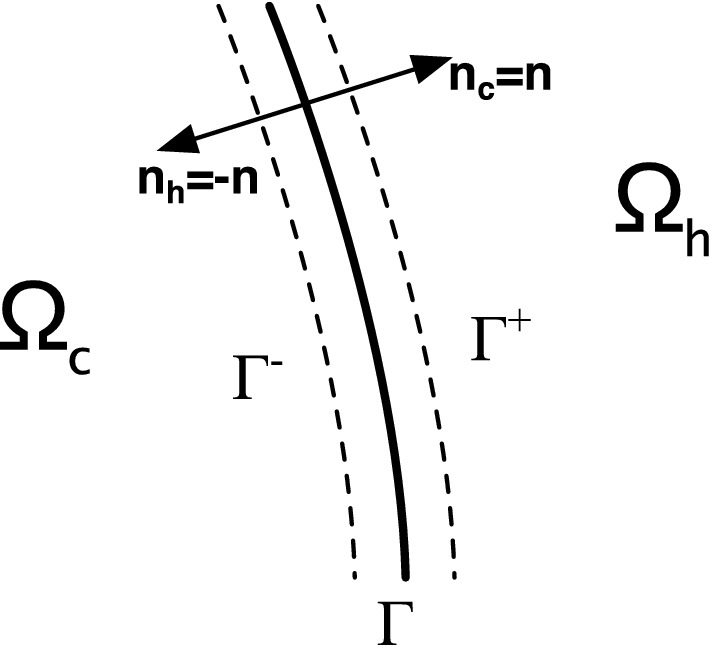
Orientation of the interface between the cloak domain $\Omega _c$ and host domain $\Omega _h$.

The methodology proposed has been validated numerically through FEM simulations, by coupling the the finite-element model of Eq. (7) in the domain $\Omega _c$ occupied by the metacontinuum, with that of Eq. (1) in the external domain of the hosting medium, $\Omega _h$. The domain $\Omega _c$ surrounds the object of radius $r_1$ and has an uniform thickness $\Delta =r_2-r_1$. The scattering object is a cylinder of infinite length and radius $r_1$, cloaked by an annular device of thickness $r_2 - r_1$. The incoming acoustic perturbation is produced by an isotropic point source located above the cylinder at a distance of $7.5 \, r_2$. The hosting medium is flowing from left to right. The two domains are in contact along the boundary $\Gamma$, assumed to be aerodynamically impermeable (details about the specific setup of the numerical model are given in the Supplementary Information). Equation (7), with $\mathbf{Q}=\mathbf{I}$, and Eq. (2) are Fourier-transformed, and the problem is solved in the frequency domain. The coupling between the two domains is applied by imposing continuity conditions for the acoustic field at the interface between the two media, as described in Iemma and Burghignoli^25^, Iemma^14^, and Iemma and Palma^12,23^. Assuming the orientation of the boundary $\Gamma$ pointing towards the hosting domain $\Omega _h$ (see Fig. 4), the continuity of pressure and normal acceleration at the media interface results in the following conditions

19 $$\begin{aligned} \tilde{p}_c = \tilde{p}_h, \quad \check{\varvec{\varrho }}^{-1} \nabla \tilde{p}_c \cdot \mathbf{n}= \nabla \tilde{p}_h \cdot \mathbf{n}+ \left( \mathbf{v}_0\cdot \nabla \mathbf{v}_h\right) \cdot \mathbf{n}\end{aligned}$$

where $\tilde{p}_c$ and $\tilde{p}_h$ are respectively the Fourier transform of the pressure in the cloak domain and in the host, $\check{\varvec{\varrho }}$ is the corrected inertia tensor derived from Eq. (7), and $\mathbf{n}$ is the $\Gamma$ unit normal vector. The uncorrected inertia tensor and bulk modulus are those proposed by Pendry, Schurig and Smith^10^ for the two-dimensional problem

20 $$\begin{aligned} \hat{\varvec{\varrho }}=\left( \begin{array}{cc} \frac{r}{r-r_1} &{}0\\ 0 &{} \frac{r-r_1}{r} \end{array}\right) , \quad \hat{\mathcal {K}}=\left( \frac{r_2-r_1}{r_2}\right) ^2 \frac{r}{r-r_1} \end{aligned}$$

**Figure 5:**
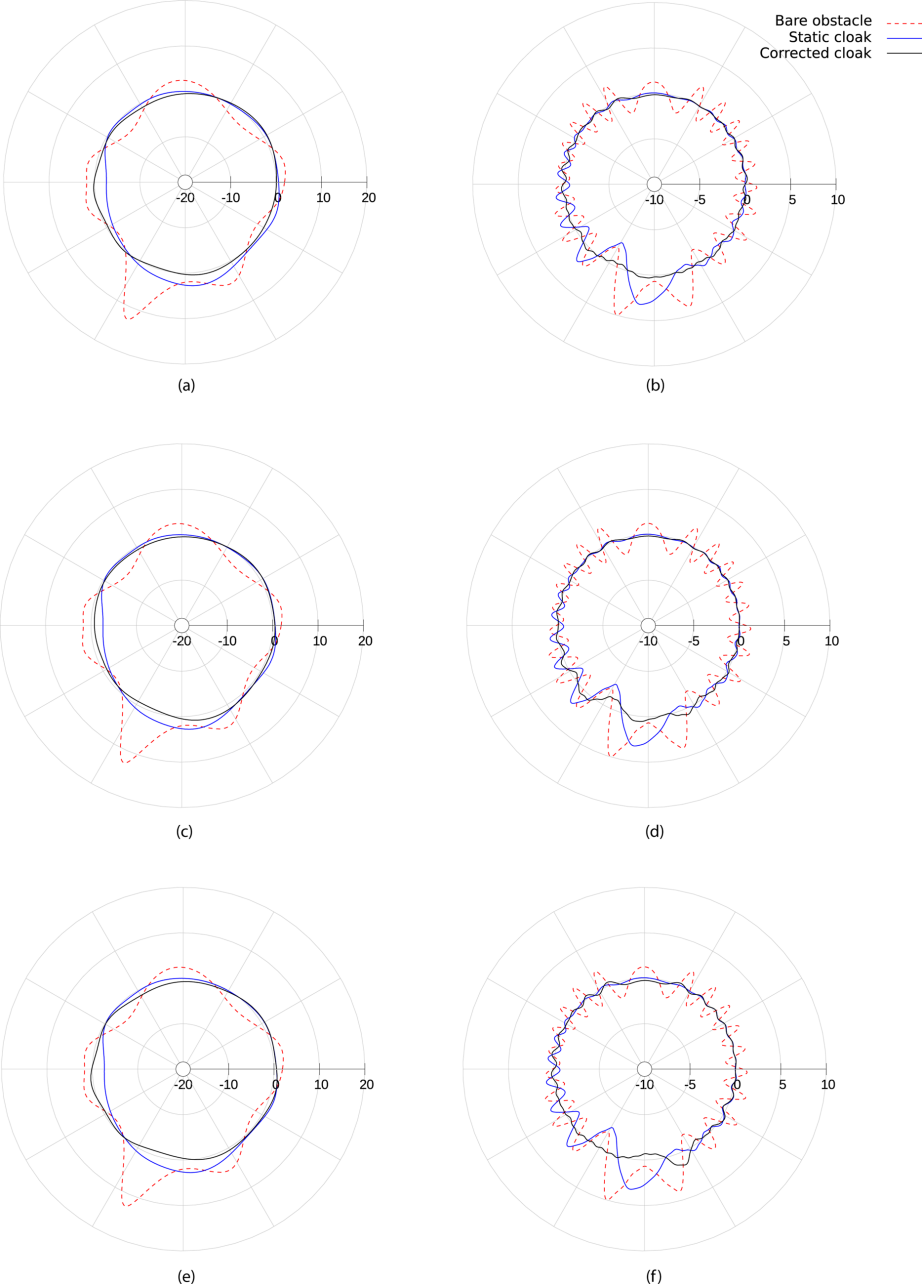
Insertion Loss directivity at $M_\infty =0.3$ for $kr_2=4$ in the near field ($r^N_{mic}=1.5 r_2$, (**a**), (**c**) and (**e**)), and in the far field ($r^F_{mic}=20 r_2$, (**b**),(**d**) and (**f**)). $\varvec{\Lambda }_{\mathrm{T}}$ is used in (**a**) and (**b**), $\varvec{\Lambda }_{\mathrm{TUS}}$ in (**c**) and (**d**), and $\varvec{\Lambda }_{\mathrm{PG}}$ in (**e**) and (**f**). Radial scale expressed in dB.

**Figure 6:**
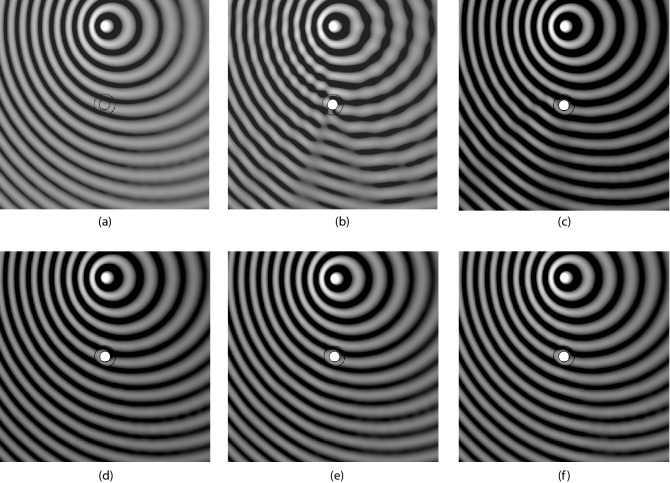
Acoustic pressure, near field visualizations at $M_\infty =0.3$ for $kr_2=4$. (**a**) shows the undisturbed perturbation, (**b**) the scattering from the bare cylinder, and (**c**) the effect of a static cloak. $\varvec{\Lambda }_{\mathrm{T}}$ correction is used in (**d**), $\varvec{\Lambda }_{\mathrm{TUS}}$ in (**e**), and $\varvec{\Lambda }_{\mathrm{PG}}$ in (**f**).

**Figure 7:**
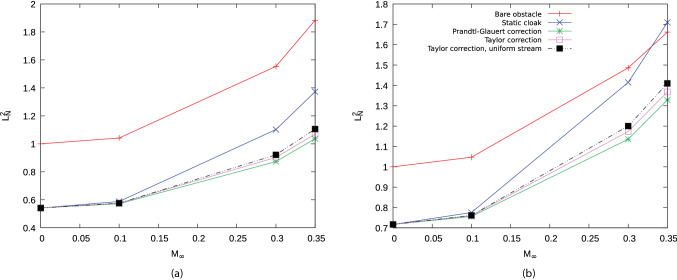
Normalized $L^2$-norm (Eq. (17)) as a function of $M_\infty$ for $kr_2=2$ (**a**), $kr_2=4$ (**b**). The scattering norm of the bare obstacle is compared with those calculated for the cloaked object. Static cloak is depicted by blue marks, whereas pink, black and green marks refer to $\varvec{\Lambda }_{\mathrm{T}}$, $\varvec{\Lambda }_{\mathrm{TUS}}$, and $\varvec{\Lambda }_{\mathrm{PG}}$, respectively.

## Supplementary information

Supplementary Information.

## Data Availability

The link to the repository of the setup files used for the simulations presented in this paper is included in the Supplementary Information.
