# Supplementary material for: Design of metacontinua in the aeroacoustic spacetime
Source: Sci Rep. 2020 Oct 23;10:18192. doi: 10.1038/s41598-020-74304-5 (PMC7584669; doi:10.1038/s41598-020-74304-5)
Supplement: Supplementary file 1 — Supplementary Information. [file 41598_2020_74304_MOESM1_ESM.pdf]

Supplementary Information

Design of Metacontinua in the Aeroacoustic Spacetime

Umberto Iemma and Giorgio Palma

Roma Tre University
Department of Engineering
via Vito Volterra, 62
00146, Rome, Italy

Corresponding author e-mail: umberto.iemma@uniroma3.it

Spacetime metric of governing equations

Equation for the acoustic potential

The relativistic structure of the equations governing the propagation of an acoustic disturbance in a flowing medium was first noticed by Unruh, W.[1,2] and Visser, M.[3,4] A thorough analysis of the relativistic spacetime structure for a barotropic fluid is presented in Visser, M. and Molina–Paris, C.[5]. Here, only the derivation of the Lorentzian metric for the full–potential convective equation is briefly outlined in support of the interested reader, with no details about the underlying aerodynamics. The propagation of an acoustic disturbance in an inviscid moving medium can be written in terms of acoustic velocity potential φ as

$$-\partial_t \left[\frac{\rho_0}{c_0^2}\left(\partial_t \varphi + \mathbf{v}\cdot\nabla\varphi\right)\right] + \nabla\cdot\left[\rho_0 \nabla\varphi - \frac{\rho_0}{c_0^2}\mathbf{v}\left(\partial_t\varphi + \mathbf{v}\cdot\nabla\varphi\right)\right] = S \tag{S1}$$

The above equation is obtained under the assumption of potential aerodynamic velocity field $\mathbf{v} = \nabla\Phi$. Under these assumptions, the dependence of the local values of density and speed of sound on the aerodynamic potential Φ can be obtained by coupling the Bernoulli's equation with the isentropic relationships between pressure, density and enthalpy. For a steady flow yields

$$\frac{c_0^2}{c_\infty^2} = 1 - \frac{\gamma-1}{c_\infty^2}\frac{\mathrm{v}^2}{2}, \qquad \frac{\rho_0}{\rho_\infty} = \left(\frac{c_0}{c_\infty}\right)^{\frac{2}{\gamma-1}} \tag{S2}$$

where γ is the ratio of specific heats, and c_∞ and ρ_∞ are the values of density and speed of sound of the asymptotic free stream. Referring to components in the four–dimensional spacetime with greek indices running from 0 to 3, and to those in the the Euclidean space with latin indices running from 1 to 3, and introducing the 4–gradient operator $\boldsymbol{\partial} \equiv (\partial_0, \partial_1, \partial_2, \partial_3) = (\partial_t/c_{\text{ref}}, \partial_i)$ and the 4–vector $\boldsymbol{\nu} = \left\{1, \mathrm{v}^1, \mathrm{v}^2, \mathrm{v}^3\right\}^T$, the spacetime form of Eq. (S1) is

$$-\boldsymbol{\partial}\cdot\left(\frac{\rho_0}{c_0^2}\boldsymbol{\nu}\boldsymbol{\nu}^T\,\boldsymbol{\partial}\varphi\right) + \boldsymbol{\partial}\left(\rho_0\,\boldsymbol{\partial}\varphi\right) = S \tag{S3}$$

which can be written as

$$\frac{1}{\sqrt{-g}}\boldsymbol{\partial}\cdot\left(\sqrt{-g}\,\boldsymbol{g}^{-1}\boldsymbol{\partial}\varphi\right) = S. \tag{S4}$$

The metric of Equation (S4) depends on the background flow as

$$\boldsymbol{g} = \frac{\rho_0}{c_0}\begin{pmatrix} -(c_0^2 - \mathrm{v}^2) & \vdots & -\mathbf{v}^T \\ \cdots & \cdot & \cdots \\ -\mathbf{v} & \vdots & \mathbf{I}\end{pmatrix}, \quad \boldsymbol{g}^{-1} = \frac{1}{\rho_0 c_0}\begin{pmatrix} -1 & \vdots & -\mathbf{v}^T \\ \cdots & \cdot & \cdots \\ -\mathbf{v} & \vdots & c_0^2\mathbf{I} - \mathbf{v}\mathbf{v}^T\end{pmatrix} \tag{S5}$$

Reformulation as inhomogeneous, uniformly–convected D'Alembertian

Manipulating Eq.(S1), the uniform convective d'Alembertian can be isolated on the left-hand side,

$$-\frac{1}{c_\infty^2}D_{tt}^\infty\varphi + \nabla^2\varphi = \frac{1}{c_\infty^2}(\sigma_1 + \sigma_2) + \frac{c_0^2}{\rho_0 c_\infty^2}(\sigma_3) \tag{S6}$$

with

$$\sigma_1 = 2\mathbf{u}\cdot\partial_t\nabla\varphi + \mathbf{U}\cdot\nabla(\mathbf{u}\cdot\nabla\varphi) + \mathbf{u}\cdot\nabla(\mathbf{v}\cdot\nabla\varphi) \tag{S7}$$

$$\sigma_2 = \nabla^2\varphi\left(c_\infty^2 - c_0^2\right) + \partial_t\mathbf{v}\cdot\nabla\varphi + D_t\varphi\,(\nabla\cdot\mathbf{u}) \tag{S8}$$

$$\sigma_3 = D_t\left(\frac{\rho_0}{c_0^2}\right)D_t\varphi - \nabla\varphi\cdot\nabla\rho_0 \tag{S9}$$

where $D_t^\infty = \partial_t + U_\infty\partial_1$ and $D_t = \partial_t + \mathbf{v}\cdot\nabla$. When $\boldsymbol{\Lambda}_{\text{PG}}$ is applied to Eq. (S6) the terms (S7) on the right–hand side can be considered as error sources that perturb the ideally cloaked field.

Reformulation as inhomogeneous $\mathscr{O}(M_\infty)$ convective wave equation

The same analysis can be performed for the transformation $\mathbf{\Lambda}_\mathrm{T}$. Equation (S1) is rewritten isolating on the left-hand side the convective d'Alembertian with non uniform velocity $\mathbf{v}$:

$$-\frac{1}{c_\infty^2}D_{tt}\varphi+\nabla^2\varphi=\frac{c_0^2}{\rho_0 c_\infty^2}\left[D_t\varphi\left(\partial_t\left(\frac{\rho_0}{c_0^2}\right)+\nabla\cdot\left(\frac{\rho_0}{c_0^2}\mathbf{v}\right)\right)+\nabla\varphi\cdot\nabla\rho_0\right]+\frac{1}{c_\infty^2}\left[\left(c_\infty^2-c_0^2\right)\nabla^2\varphi-\partial_t\mathbf{v}\cdot\nabla\varphi\right] \tag{S10}$$

Observing that $D_{tt}=\partial_{tt}+2\mathbf{v}\cdot\partial_t\nabla+\mathbf{v}\cdot\nabla\left(\mathbf{v}\cdot\nabla\right)$, the $\mathscr{O}(M_\infty)$ approximation of the convective operator in Eq.(S10) gives

$$-\frac{1}{c_\infty^2}\left(\partial_{tt}\varphi+2\mathbf{v}\cdot\partial_t\varphi\nabla\varphi\right)+\nabla^2\varphi=\frac{c_0^2}{\rho_0 c_\infty^2}\left(\sigma_4\right)+\frac{1}{c_\infty^2}\left(\sigma_5\right) \tag{S11}$$

with

$$\sigma_4=D_t\left(\partial_t\left(\frac{\rho_0}{c_0^2}\right)+\nabla\cdot\left(\frac{\rho_0}{c_0^2}\mathbf{v}\right)\right)+\nabla\varphi\cdot\nabla\rho_0 \tag{S12}$$

$$\sigma_5=\left(c_\infty^2-c_0^2\right)\nabla^2\varphi-\partial_t\mathbf{v}\cdot\nabla\varphi-\mathbf{v}\cdot\nabla\left(\mathbf{v}\cdot\nabla\varphi\right) \tag{S13}$$

Spacetime equation for the metacontinuum

The most general form of equation governing the propagation of an acoustic disturbance within a metafluid has the form (see Norris[6,7])

$$-\partial_{tt}p+c_{\mathrm{ref}}^2\,\hat{\mathscr{K}}\,\mathbf{Q}\colon\nabla\left(\hat{\boldsymbol{\rho}}^{-1}\mathbf{Q}\,\nabla p\right)=0 \tag{S14}$$

where ρ_{ref}, $\mathscr{K}_{\mathrm{ref}}$, and $c_{\mathrm{ref}}=\sqrt{\mathscr{K}_{\mathrm{ref}}/\rho_{\mathrm{ref}}}$ are the reference density, bulk modulus, and speed of sound, respectively. Recalling the tensor differential identity $\nabla\cdot\left(\mathbf{S}^T\mathbf{w}\right)=\mathbf{S}\colon\nabla\mathbf{w}+\mathbf{w}\cdot\left(\nabla\cdot\mathbf{S}\right)$ where $\mathbf{S}$ is a generic second–order tensor and $\mathbf{w}$ is a vector (see, *e.g.*, Gurtin[8]), and applying this identity to Eq. (S14), with $\mathbf{S}=\mathbf{Q}$ and $\mathbf{w}=\hat{\boldsymbol{\rho}}^{-1}\mathbf{Q}\,\nabla p$, yields

$$-\partial_{tt}p+c_{\mathrm{ref}}^2\,\hat{\mathscr{K}}\,\nabla\cdot\left(\mathbf{Q}\,\boldsymbol{\rho}^{-1}\mathbf{Q}\,\nabla p\right)=0 \tag{S15}$$

Equation (S15) can be easily recast in spacetime, generalised form as

$$\sqrt{\frac{\hat{\rho}}{\hat{\mathscr{K}}Q^2}}\,\boldsymbol{\partial}\cdot\left(\sqrt{\frac{\hat{\mathscr{K}}Q^2}{\hat{\rho}}}\,\hat{\boldsymbol{g}}^{-1}\,\boldsymbol{\partial}p\right)=0. \tag{S16}$$

where

$$\hat{\boldsymbol{g}}=\sqrt{\frac{\hat{\mathscr{K}}Q^2}{\hat{\rho}}}\begin{pmatrix}-1 & \vdots & 0\\ \cdots & \cdot & \cdots\\ 0 & \vdots & \mathbf{Q}^{-1}\,\hat{\boldsymbol{\rho}}\,\mathbf{Q}^{-1}\end{pmatrix},\quad \hat{\boldsymbol{g}}^{-1}=\sqrt{\frac{\hat{\rho}}{\hat{\mathscr{K}}Q^2}}\begin{pmatrix}-1 & \vdots & 0\\ \cdots & \cdot & \cdots\\ 0 & \vdots & \mathbf{Q}\,\hat{\boldsymbol{\rho}}^{-1}\,\mathbf{Q}\end{pmatrix}. \tag{S17}$$

Spacetime transformation metrics

This section is dedicated to some consideration about the structure of the spacetime transformations used and their relationship with the class of the relativistic Lorentzian transformations. An extensive analysis related this topic is available in Gregory, A, *et al.*[9] , where the Lorentzian differential geometry is used to reinterpret classic aeroacoustic concepts like Doppler shift and convective Green's function. Mancini, S. *et al.*[10] introduced a combination of the two transformations used here to derive a new convective integral formulation valid for weakly non–uniform flows. Assuming a background stream aligned with the first spatial direction x_1, the spacetime reformulation of the Prandtl–Glauert coordinate transformation is

$$\xi_0'=\beta\xi_0+M_\infty\frac{\xi_1}{\beta},\quad \xi_1'=\frac{\xi_1}{\beta},\quad \xi_2'=\xi_2,\quad \xi_3'=\xi_3 \tag{S18}$$

A simple manipulation of the ξ_0' expression can emphasize the relationship of Eq. (S18) with a classic Lorentz boost along the ξ_1 direction of intensity M_∞ (ξ_2' and ξ_3' remain unchanged and are not indicated for the sake of compactness). Indeed, Eq. S18 can be written as

$$\begin{cases}\xi_0'=\dfrac{1}{\beta}\left[\xi_0+M_\infty\left(\xi_1-M_\infty\xi_0\right)\right]=\dfrac{1}{\beta}\left(\xi_0+M_\infty\xi_1\right)-\dfrac{M_\infty^2}{\beta}\xi_0\\ \xi_1'=\dfrac{1}{\beta}\xi_1\end{cases} \tag{S19}$$

Its Jacobian matrix is

$$\mathbf{\Lambda}_{\rm PG} = \begin{pmatrix} \beta & M_\infty/\beta & 0 & 0 \\ 0 & 1/\beta & 0 & 0 \\ 0 & 0 & 1 & 0 \\ 0 & 0 & 0 & 1 \end{pmatrix}. \tag{S20}$$

Let's now assume that the background flow is steady, incompressible, and potential, *i.e.*, $\mathbf{v}(\mathbf{x}) = \nabla\Phi(\mathbf{x})$ with $\nabla^2\Phi = 0$. Writing the aerodynamic field as a superposition of a perturbation $\mathbf{u}(\mathbf{x})$ onto a uniform free stream, yields the aerodynamic velocity potential $\Phi(\mathbf{x}) = U_\infty x_1 + \phi(\mathbf{x}) = U_\infty \xi_1 + \phi(\boldsymbol{\xi})$, with $\mathbf{u} = \nabla\phi$. The Taylor transformation has the form

$$\xi_0' = \xi_0 + M_\infty \hat{\Phi}(\mathbf{x}) = \xi_0 + M_\infty \left(\xi_1 + \hat{\phi}\right), \qquad \xi_i' = \xi_i \tag{S21}$$

where $\hat{\bullet}$ indicates normalization by $\|U_\infty\|$. Transformation S21 maps the $\mathscr{O}(M_\infty)$ convective wave equation into the steady d'Alembertian. The transformation matrix is

$$\mathbf{\Lambda}_{\rm T} = \begin{pmatrix} 1 & M_\infty\left(1+\partial_1\hat{\phi}\right) & M_\infty\partial_2\hat{\phi} & M_\infty\partial_3\hat{\phi} \\ 0 & 1 & 0 & 0 \\ 0 & 0 & 1 & 0 \\ 0 & 0 & 0 & 1 \end{pmatrix} \tag{S22}$$

As already noticed, $\mathbf{\Lambda}_{\rm PG}$ maps the convective equation into the static one for a uniform aerodynamics and with no limitations on the value of M_∞, whereas $\mathbf{\Lambda}_{\rm T}$ can handle non–uniformity of the flow, but only under the assumption of low Mach number. Nevertheless, they are closely related. Indeed, we can write

$$\mathbf{\Lambda}_{\rm PG} = \mathbf{\Lambda}_{\rm T} - \begin{pmatrix} 0 & M_\infty\partial_1\hat{\phi} & M_\infty\partial_2\hat{\phi} & M_\infty\partial_3\hat{\phi} \\ 0 & 0 & 0 & 0 \\ 0 & 0 & 0 & 0 \\ 0 & 0 & 0 & 0 \end{pmatrix} + \mathscr{O}(M_\infty^2) \tag{S23}$$

to emphasize the fact that the two transformations coincide to the order $\mathscr{O}(M_\infty)$ when $\mathbf{u}(\mathbf{x}) = 0\ \forall\mathbf{x} \in \Omega_h$.

Finite element simulations

The numerical simulations presented in the article are performed with the FEM software COMSOL Multiphysics®[11]. All the analyses are performed under a two-dimensional approximation assuming a monochromatic, periodic acoustic perturbation that propagates in an inviscid, compressible medium where a steady, potential flow evolves. The numerical setup has been geometrically parametrized with respect to the radius of the scattering cylinder r_1. A square domain with side $l = 120\,r_1$ is used for the aerodynamic calculations $\mathscr{D}_a$, while a concentric circle of radius $r_d = 60\,r_1$ defines the aeroacoustic domain $\mathscr{D}_{ac}$. A circle of radius $r_2 = 2\,r_1$ placed in the center of the above defined domains delimits the cloak domain $\mathscr{D}_c$. In order to facilitate the interest reader in the reproduction of the results presented, a detailed description of the numerical setup is given in the following. The *Compressible Potential Flow* module is used to solve for the aerodynamic field in the hosting fluid domain. A *Slip Velocity* condition is set on the cloak boundary, hence imposing its aerodynamic impermeability. A *Mass Flow* condition is set at the inlet boundary, and at the outlet boundary a *Normal Flow* condition is applied.

The *Linearized Potential Flow* module is used for the aeroacoustic calculations in the hosting fluid domain. The module solves for the acoustic potential φ in the frequency domain, solving the following equation

$$-i\omega\left[\frac{\rho_0}{c_0^2}\left(i\omega\tilde{\varphi} + \mathbf{v}\cdot\nabla\tilde{\varphi}\right)\right] + \nabla\cdot\left[\rho_0\nabla\varphi - \frac{\rho_0}{c_0^2}\mathbf{v}\left(i\omega\tilde{\varphi} + \mathbf{v}\cdot\nabla\varphi\right)\right] = \tilde{S} \tag{S24}$$

The solution from the aerodynamic calculations is considered as a background flow for the aeroacoustic module. The aerodynamic solution is hence coupled with the aeroacoustic module, setting the resulting velocity, density and speed of sound field distribution of the first as values for the background flow of the latter with the *Multiphysics* coupling. The *Coefficient form PDE* module is used to model the acoustic propagation inside the metamaterial domain ω_c. This module solves a second–order partial differential equation in the frequency domain of the general form

$$-\omega^2 e_a\tilde{u} + i\omega d_a\tilde{u} + \nabla\cdot\left(-\mathbf{C}\nabla\tilde{u} - \boldsymbol{\alpha}\tilde{u} + \boldsymbol{\gamma}\right) + \boldsymbol{\beta}\cdot\nabla\tilde{u} + a\,\tilde{u} = \tilde{f}. \tag{S25}$$

For the simulations presented here, the variable u is representative of p_c when, defining $W^{\mu\nu}$ as the components of $\mathbf{W} = \sqrt{-\breve{g}}\breve{\mathbf{g}}^{-1}$, the coefficients of Eq.(S25) assume the values

$$e_a = d_a = \boldsymbol{\gamma} = f = 0$$

and

$$\mathbf{C} = \boldsymbol{\rho}^{-1} = \begin{Bmatrix} W^{11} & W^{12} \\ W^{21} & W^{22} \end{Bmatrix}, \quad \boldsymbol{\alpha} = \begin{Bmatrix} W^{01} \\ W^{02} \end{Bmatrix} \frac{i\omega}{c_0}, \quad \boldsymbol{\beta} = -\begin{Bmatrix} W^{01} \\ W^{02} \end{Bmatrix} \frac{i\omega}{c_0}, \quad a = \frac{\omega^2}{c_0^2} W^{00}. \tag{S26}$$

The boundaries of the scattering object are considered to be acoustically rigid imposing the *Sound Hard Boundary* condition. The cloak and the hosting fluid are coupled at their interface using a Dirichlet condition in the PDE module to impose the continuity of pressure, $p_c = p_h$, and a Neumann-type condition in the linearized potential flow module to guarantee continuity of the normal acoustic acceleration at the boundary Γ (see Fig. 1). The frequency domain form of the momentum equations in Ω_c yields the following condition for the normal acoustic velocity along Γ

$$\mathbf{v}_h \cdot \mathbf{n}_h = v_{hn} = -\boldsymbol{\rho}^{-1} \nabla p \cdot \mathbf{n}_c = -((W^{11}\,\partial_x p_c + W^{12}\,\partial_y p_c)\,n_x + (W^{21}\,\partial_x p_c + W^{22}\,\partial_y p_c)\,n_y)/(i\omega\rho_0). \tag{S27}$$

The acoustic source is created with a *Mass Flow Point Source* of unit intensity inside the hosting fluid. To mimic an open domain, at the outer boundaries of $\mathscr{D}_{ac}$ a *Plane Wave Radiation* condition is imposed. The calculation domain is meshed using second order Lagrangian triangular elements, with representative size $\lambda/20 < h < \lambda/10$. A minimum number of elements is imposed at the cylinder and cloak boundaries, $N \geq 15\pi r_2/\lambda_{max}$ in order to have at least 7 elements per wavelength on their perimeter at the highest considered frequency. Eight layers of thin quadrangular elements are placed over the interface between the hosting and the cloaking domains, exploiting the *Boundary Layer* meshing tool of COMSOL, This meshing strategy proved to improve the correct imposition of the boundary conditions, and hence the continuity of the solution across the domains interface.

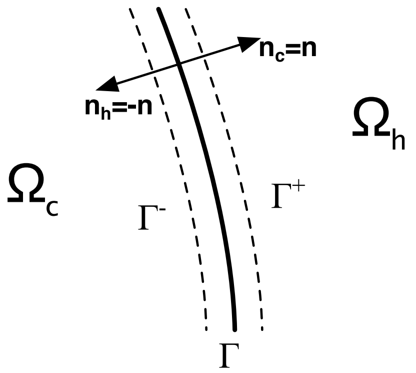

Figure 1. Nomenclature of the interface between the cloak domain Ω_c and host domain Ω_h.

The setup files of the COMSOL Multiphysics® simulations are freely available on the Zenodo repository at the URL https://doi.org/10.5281/zenodo.3838233.

Supplementary results

In order to give a better overview of the effectiveness of the spacetime corrections, the complete set of results of the numerical simulations is reported here. Figures 2, 3 and 4 show the near pressure field around the scattering cylinder at $M_\infty = 0.1$, $M_\infty = 0.3$, and $M_\infty = 0.35$, respectively. In all these figures the free field is shown as the ideal reference for a perfect acoustic cloaking, and the comparison between the effect of the bare object (b), the object cloaked with the static metamaterial (c) and the corrected devices obtained with the three spacetime transformations, $\boldsymbol{\Lambda}_{\mathrm{T}}$ (d), $\boldsymbol{\Lambda}_{\mathrm{TUS}}$ (e) and $\boldsymbol{\Lambda}_{\mathrm{PG}}$ (f), is shown at the lowest simulated frequency, $kr_2 = 1$. Similarly, Figs. 5, 6 and 7 report simulations at $kr_2 = 2$, and Figs. 8, 9 and 10 show the results for the highest simulated frequency, $kr_2 = 4$.

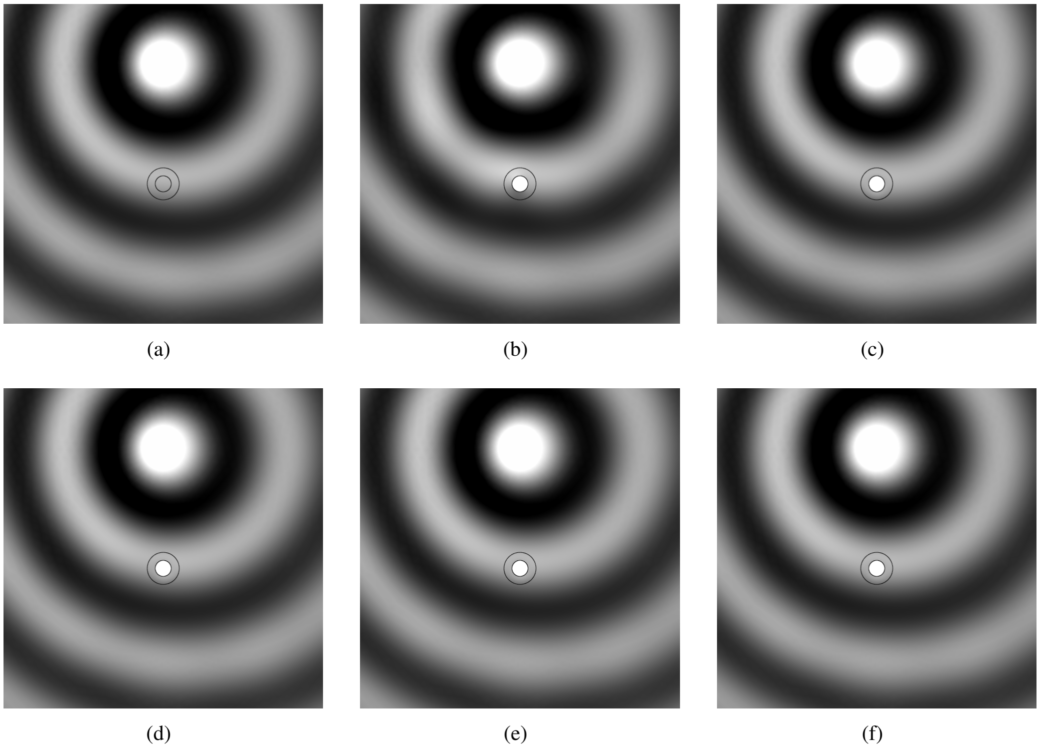

Figure 2. Field visualizations, $kr_2 = 1$, $M_\infty = 0.1$: (a) free field, (b) bare cylinder, (c) static design, (d) $\mathbf{\Lambda}_\mathrm{T}$ correction, (e) $\mathbf{\Lambda}_\mathrm{TUS}$ correction and (f) $\mathbf{\Lambda}_\mathrm{PG}$ correction.

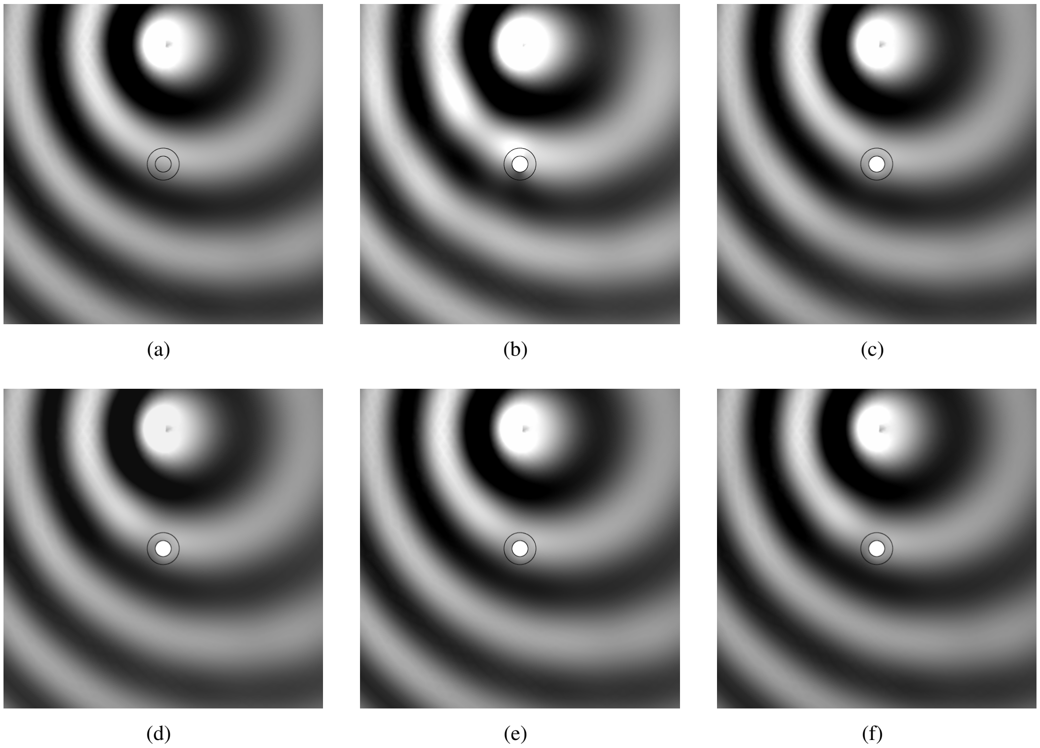

Figure 3. Field visualizations, $kr_2 = 1$, $M_\infty = 0.3$: (a) free field, (b) bare cylinder, (c) static design, (d) $\mathbf{\Lambda}_\mathrm{T}$ correction, (e) $\mathbf{\Lambda}_\mathrm{TUS}$ correction and (f) $\mathbf{\Lambda}_\mathrm{PG}$ correction.

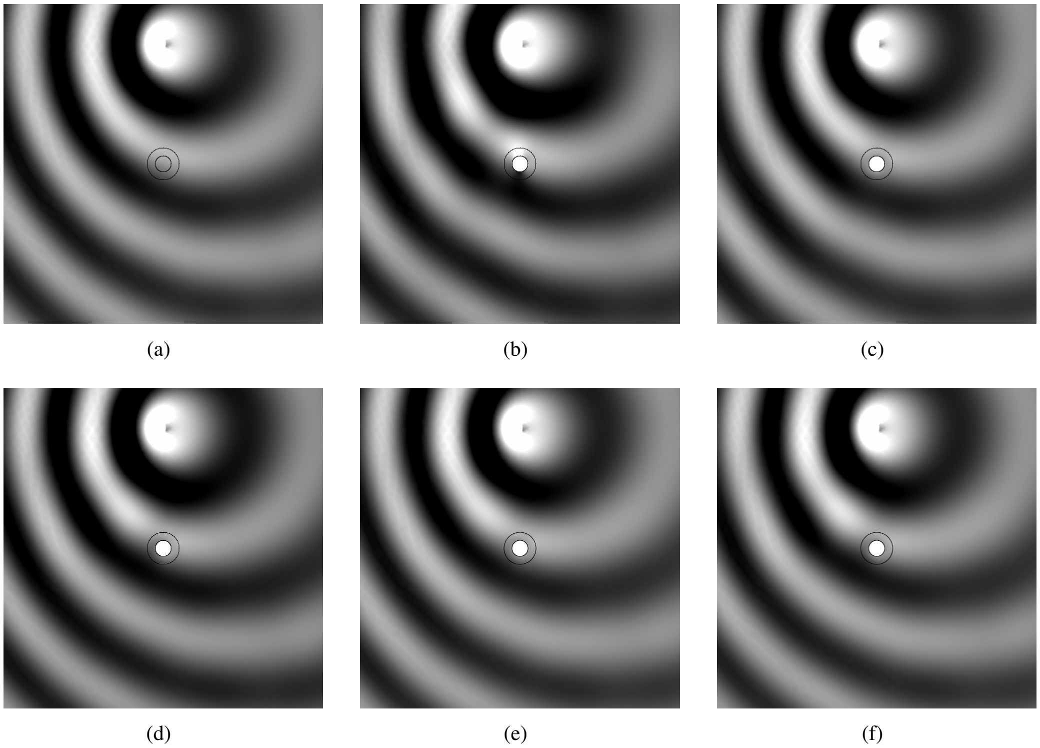

Figure 4. Field visualizations, $kr_2 = 1$, $M_\infty = 0.35$: (a) free field, (b) bare cylinder, (c) static design, (d) $\mathbf{\Lambda}_{\mathrm{T}}$ correction, (e) $\mathbf{\Lambda}_{\mathrm{TUS}}$ correction and (f) $\mathbf{\Lambda}_{\mathrm{PG}}$ correction.

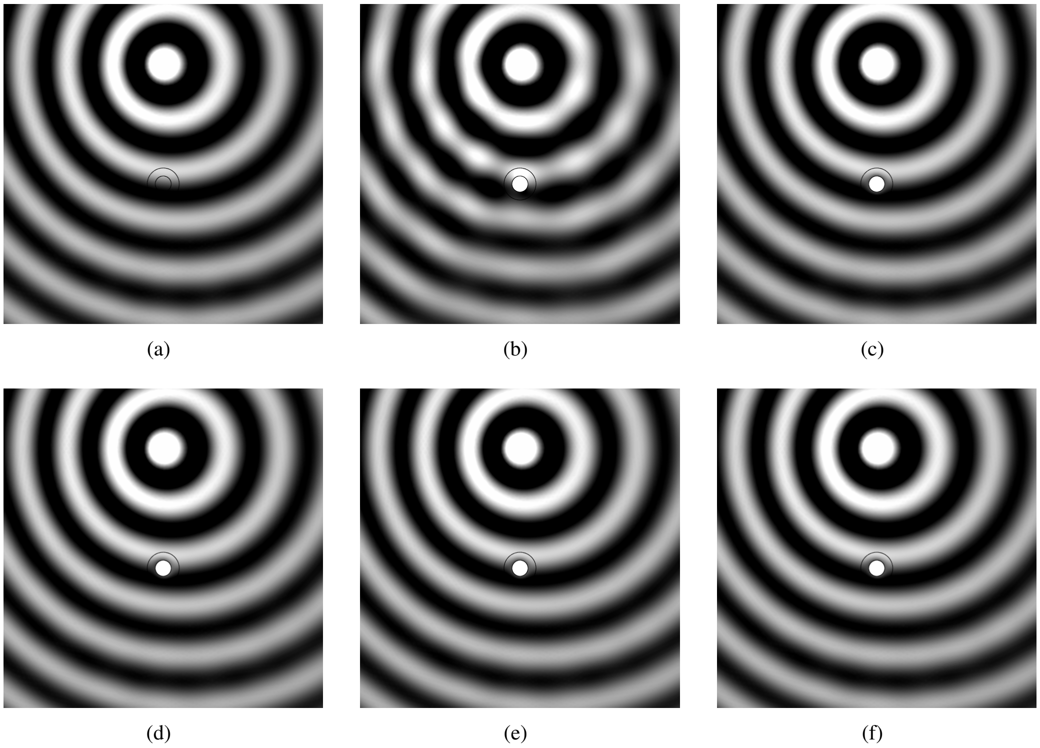

Figure 5. Field visualizations, $kr_2 = 2$, $M_\infty = 0.1$: (a) free field, (b) bare cylinder, (c) static design, (d) $\mathbf{\Lambda}_{\mathrm{T}}$ correction, (e) $\mathbf{\Lambda}_{\mathrm{TUS}}$ correction and (f) $\mathbf{\Lambda}_{\mathrm{PG}}$ correction.

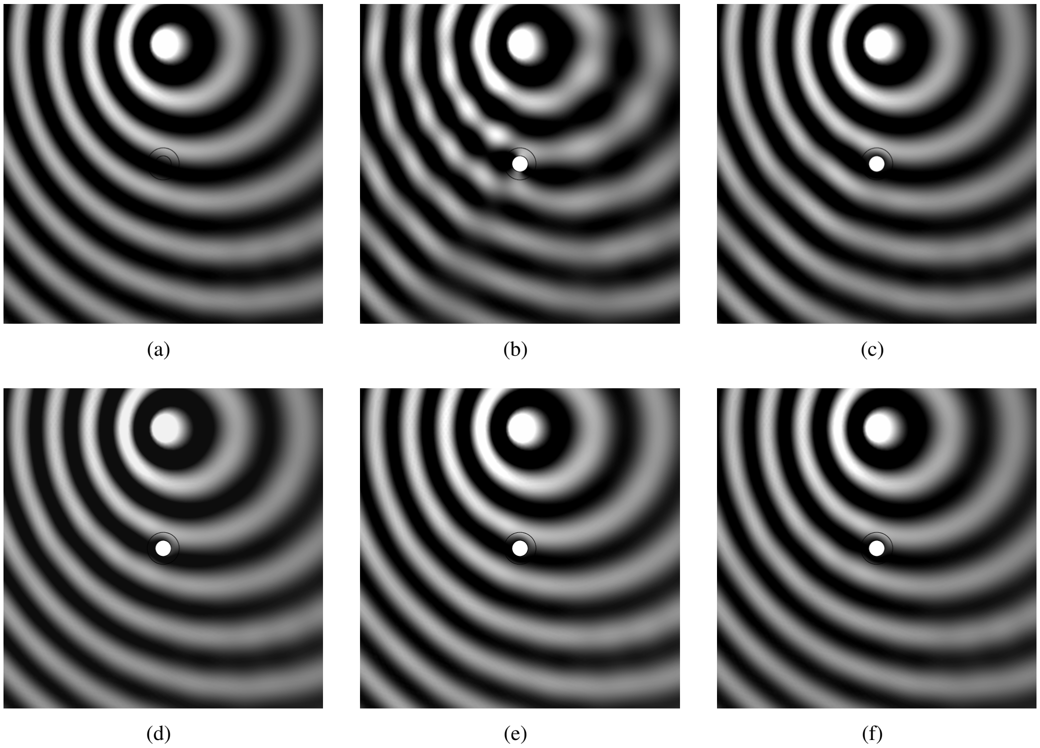

Figure 6. Field visualizations, $kr_2 = 2$, $M_\infty = 0.3$: (a) free field, (b) bare cylinder, (c) static design, (d) $\mathbf{\Lambda}_{\mathrm{T}}$ correction, (e) $\mathbf{\Lambda}_{\mathrm{TUS}}$ correction and (f) $\mathbf{\Lambda}_{\mathrm{PG}}$ correction.

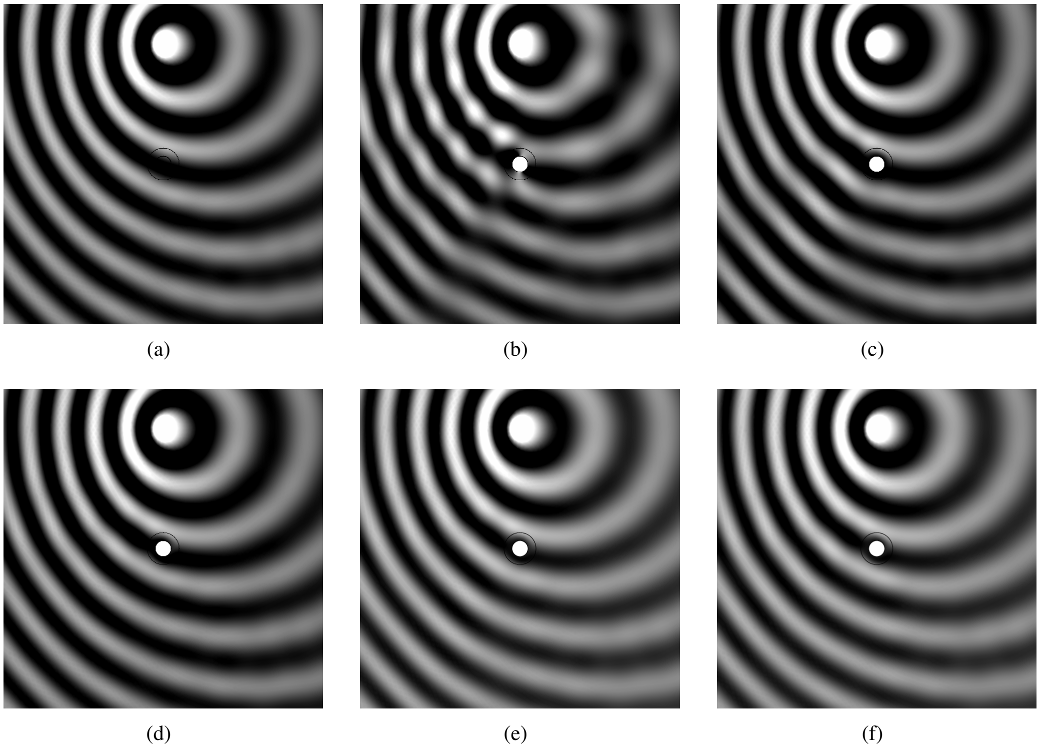

Figure 7. Field visualizations, $kr_2 = 2$, $M_\infty = 0.35$: (a) free field, (b) bare cylinder, (c) static design, (d) $\mathbf{\Lambda}_{\mathrm{T}}$ correction, (e) $\mathbf{\Lambda}_{\mathrm{TUS}}$ correction and (f) $\mathbf{\Lambda}_{\mathrm{PG}}$ correction.

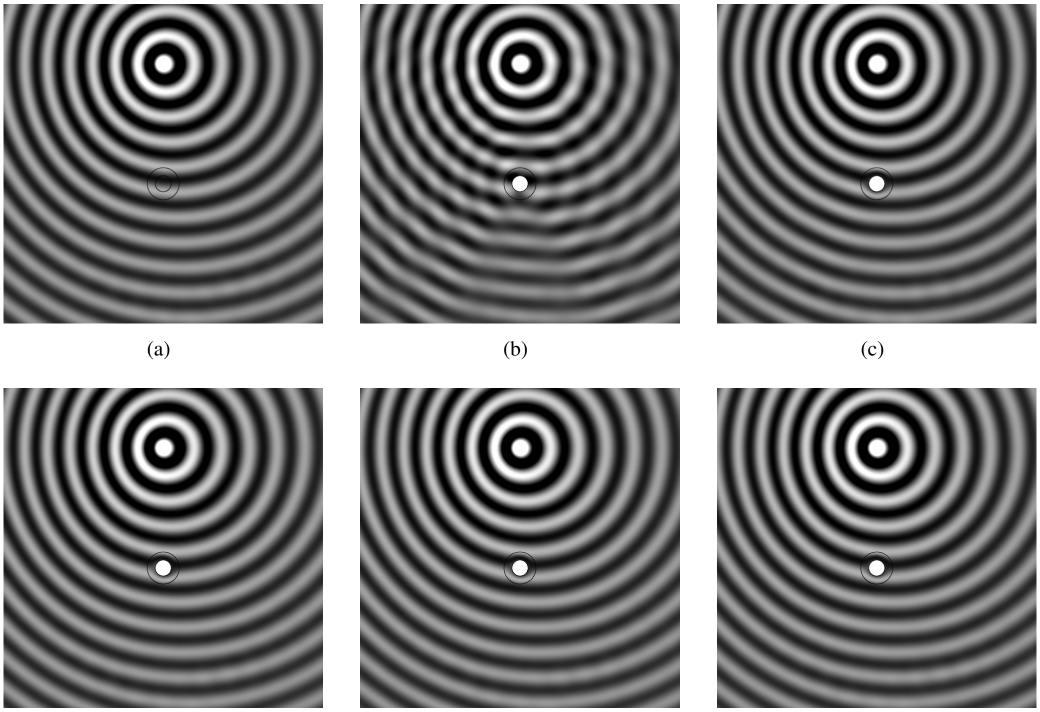

(a) (b) (c)

(d) (e) (f)

Figure 8. Field visualizations, $kr_2 = 4$, $M_\infty = 0.1$: (a) free field, (b) bare cylinder, (c) static design, (d) $\mathbf{\Lambda}_{\mathrm{T}}$ correction, (e) $\mathbf{\Lambda}_{\mathrm{TUS}}$ correction and (f) $\mathbf{\Lambda}_{\mathrm{PG}}$ correction.

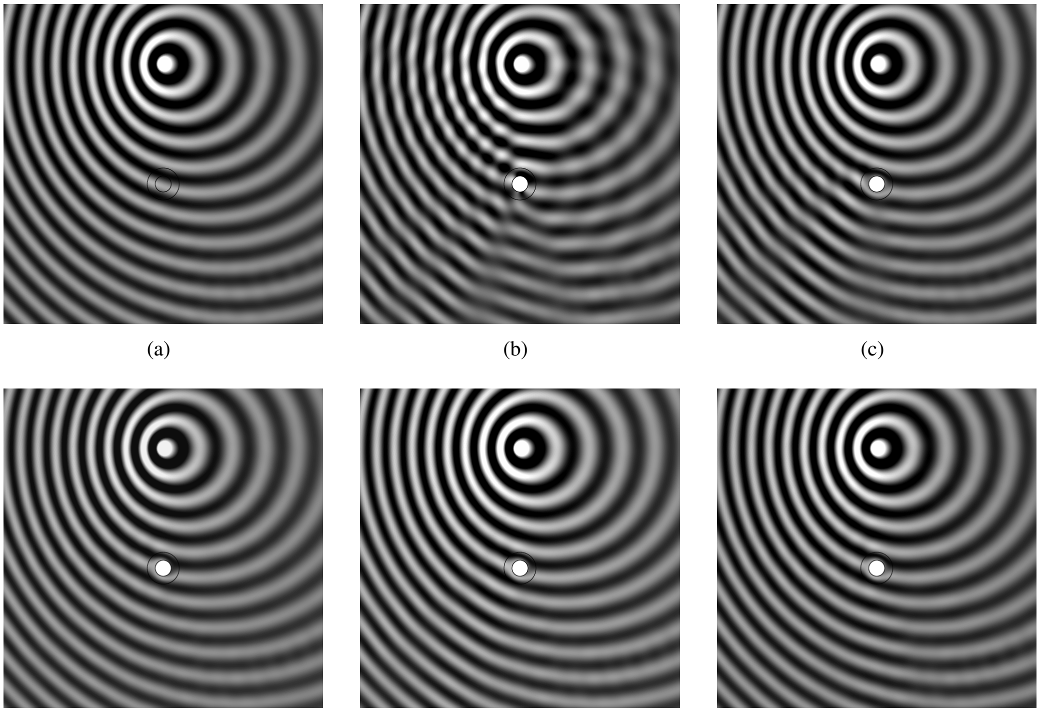

(a) (b) (c)

(d) (e) (f)

Figure 9. Field visualizations, $kr_2 = 4$, $M_\infty = 0.3$: (a) free field, (b) bare cylinder, (c) static design, (d) $\mathbf{\Lambda}_{\mathrm{T}}$ correction, (e) $\mathbf{\Lambda}_{\mathrm{TUS}}$ correction and (f) $\mathbf{\Lambda}_{\mathrm{PG}}$ correction.

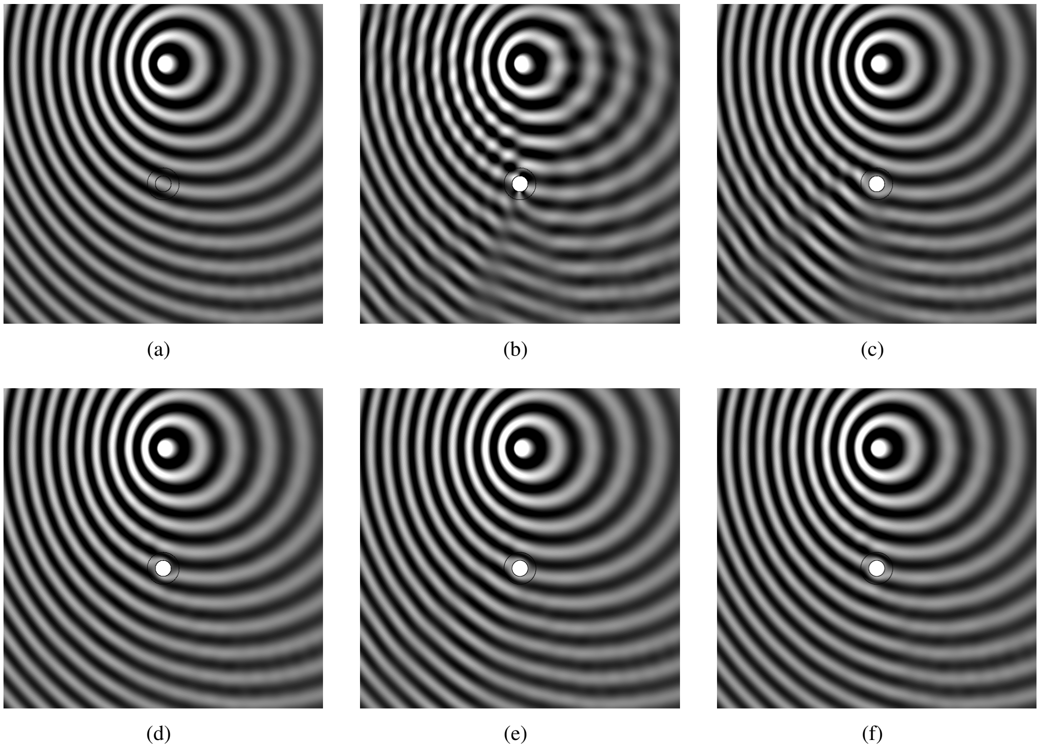

Figure 10. Field visualizations, $kr_2 = 4$, $M_\infty = 0.35$: (a) free field, (b) bare cylinder, (c) static design, (d) $\mathbf{\Lambda}_\mathrm{T}$ correction, (e) $\mathbf{\Lambda}_\mathrm{TUS}$ correction and (f) $\mathbf{\Lambda}_\mathrm{PG}$ correction.

References

1. Unruh, W. G. Experimental black-hole evaporation? *Phys. Rev. Lett.* **46**, 1351–1353 (1981).
2. Unruh, W. G. Sonic analogue of black holes and the effects of high frequencies on black hole evaporation. *Phys. Rev. D* **51**, 2827–2838 (1995).
3. Visser, M. Acoustic propagation in fluids: an unexpected example of lorentzian geometry (1993).
4. Visser, M. Acoustic black holes: horizons, ergospheres and Hawking radiation. *Classical and Quantum Gravity* **15**, 1767 (1998).
5. Visser, M. & Molina-París, C. Acoustic geometry for general relativistic barotropic irrotational fluid flow. *New Journal of Physics* **12** (2010).
6. Norris, A. N. Acoustic cloaking theory. *Proceedings of the Royal Society A: Mathematical, Physical and Engineering Sciences* **464**, 2411–2434 (2008).
7. Norris, A. N. Acoustic metafluids. *The Journal of the Acoustical Society of America* **125**, 839–849 (2009).
8. Gurtin, M. *An Introduction to Continuum Mechanics*. Mathematics in Science and Engineering (Elsevier Science, 1982).
9. Gregory, A. L., Sinayoko, S., Agarwal, A. & Lasenby, J. An acoustic spacetime and the Lorentz transformation in aeroacoustics. *International Journal of Aeroacoustics* **14**, 37 (2014).
10. Mancini, S., Astley, R. J., Sinayoko, S., Gabard, G. & Tournour, M. An integral formulation for wave propagation on weakly non-uniform potential flows. *Journal of Sound and Vibration* **385**, 184–201 (2016).
11. Comsol multiphysics® v5.4. www.comsol.com, COMSOL AB, Stockholm, Sweden (2019).
